# Subjective Perception of Recovery and Measured Olfactory Function in COVID-19 Patients

**DOI:** 10.3390/v15071418

**Published:** 2023-06-23

**Authors:** Emilia Cancellieri, Anna Kristina Hernandez, Helena Degkwitz, Elisabeth Kahre, Judith Blankenburg, Theresa S. Horst, Paula Czyborra, Paolo Boscolo-Rizzo, Thomas Hummel

**Affiliations:** 1 Smell & Taste Clinic, Department of Otorhinolaryngology, Faculty of Medicine Carl Gustav Carus, Technische Universität Dresden, 01307 Dresden, Germany; 2 Department of Medical, Surgical and Health Sciences, Section of Otolaryngology, University of Trieste, 34149 Trieste, Italy; 3 Department of Otolaryngology, Head and Neck Surgery, Philippine General Hospital, University of the Philippines Manila, Manila 1000, Philippines; 4 Department of Otolaryngology, Head and Neck Surgery, Asian Hospital and Medical Center, Muntinlupa 1780, Philippines; 5 Department of Pediatric Medicine, Technische Universität Dresden, 01307 Dresden, Germany

**Keywords:** smell, olfaction disorders, COVID-19, sensory threshold, self-report, parosmia

## Abstract

This cross-sectional study aimed to investigate self-rated olfactory dysfunction in relation to measured olfactory function after partial or complete subjective recovery in individuals with a history of coronavirus disease 2019 (COVID-19) infection. A total of 186 individuals (aged 5–62 years) with a history of COVID-19 infection were included. Visual analogue scale (VAS) ratings for olfactory function (before, during, and after infection) and age-appropriate psychophysical olfactory test scores (odor threshold and odor identification: “Sniffin’ Sticks” for adults and both “Sniffin’ Sticks” and “U-Sniff” for children) were determined. Participants were assigned to four “age groups” and three “recovery classes” (incomplete recovery, complete recovery, no smell loss). Surprisingly, there were no significant differences in odor threshold and adult identification scores between the “recovery classes”. However, children with “incomplete recovery” had lower identification scores than those with “complete recovery” (*p* = 0.033) and those with “no smell loss” (*p* = 0.022). The pediatric age groups had significantly higher VAS ratings during and after COVID-19 compared to older participants. Older individuals experienced greater magnitude of changes in their sense of smell after COVID-19 infection, but those with parosmia were 3.5 times more likely to report “incomplete recovery" of olfaction after COVID-19. The general prognosis for olfactory recovery after COVID-19 is good but appears to be particularly confounded by the presence of parosmia, leading patients to subjectively report incomplete olfactory recovery. Although it is of high significance to monitor recovery using validated psychophysical olfactory tests, subjective measures of olfaction help provide specific insight, especially for qualitative olfactory dysfunction.

## 1. Introduction

Coronavirus disease 2019 (COVID-19) is a multisystem infectious disease that primarily affects the respiratory tract and is caused by severe acute respiratory syndrome-coronavirus-2 (SARS-CoV-2) [1]. It is now widely known that, at least for virus variants prior to the Omicron variant, olfactory dysfunction (OD) is one of the most frequent symptoms of COVID-19. An early systematic review and meta-analysis conducted on 3563 patients by Borsetto et al. showed an overall prevalence of 47% for taste and olfactory disorders among the population [2], while a subsequent systematic review and meta-analysis by Qiu et al. identified a pooled prevalence of 47% for smell dysfunction [3]. It is also known that the prevalence of olfactory loss differs between older and younger people. Olfactory dysfunction is the most common otorhinolaryngological COVID-19 symptom in the general population [3], whereas in children, olfactory dysfunction only occurs in 8% [4].

Various methods can be used to assess olfactory function. Psychophysical tests like the “Sniffin’ Sticks”, the University of Pennsylvania Smell Identification Test (SIT-40), and the Connecticut Chemosensory Clinical Research Center (CCCRC) test [5] have been extensively used as reliable measures of olfaction. However, given the risk for virus transmission and the strict infection precautions, particularly during the early phases of the pandemic, many studies on COVID-19-associated olfactory dysfunction used self-ratings like the Questionnaire of Olfactory Disorders or the Visual Analogue Scale (VAS) [6]. It has been previously shown that self-ratings may underestimate the prevalence of OD [7], and studies have observed differences in the prevalence of OD depending on the method of testing [5,8,9,10]. Nevertheless, numerous studies have reported a correlation between the two types of measurements (e.g., [11,12]).

This study aimed to investigate self-rated olfactory dysfunction in relation to measured olfactory function after partial or complete subjective recovery in individuals with a history of COVID-19 infection.

## 2. Materials and Methods

### 2.1. Participants

A total of 295 patients were recruited between January and June 2022 to participate in this cross-sectional study. Eligible participants were patients who consulted at the Department of Pediatric Medicine of the University Hospital Dresden and their parents.

Inclusion criteria were a history of one COVID-19 infection confirmed by a positive COVID-19 polymerase chain reaction (PCR) test and self-reported smell loss during infection. Exclusion criteria were incomplete data about COVID-19 diagnosis and incomplete olfactory assessment results. An accurate medical history was collected, including information on age, sex, and date of diagnosis, to also exclude other presumed causes and previous history of smell loss. In the same visit, participants were asked to rate their olfactory function for three time points and undergo an extensive psychophysical olfactory test.

### 2.2. Visual Analogue Scale (before, during, after COVID-19 Infection)

Patients were asked to subjectively rate their olfactory function for three time points: (1) before, (2) during, and (3) after COVID-19 infection using an 11-point VAS, ranging from 0 (no olfactory function) to 10 (excellent olfactory function).

### 2.3. Recovery Classes

Based on their VAS ratings, the participants were divided into three groups according to their subjective perception of recovery (“recovery classes”), namely: 1—“complete recovery”, for patients who reported a decrease in olfactory function during the infection but a full recovery of olfactory function after the end of illness (*n* = 57); 2—“incomplete recovery”, for patients who reported a decrease in olfactory function during the infection but not a full recovery after the end of the illness (*n* = 77); and 3—“no smell loss”, for patients who denied having olfactory loss during COVID-19 (*n* = 52). A difference of 1 point or more in the VAS ratings before and after COVID-19 was regarded as “incomplete recovery”.

### 2.4. Age Groups

The participants were also divided into four “age groups”: (A) 5–11 years (*n* = 19); (B) 12–17 years (*n* = 30); (C) 18–44 years (*n* = 78); and (D) 45–62 years (*n* = 59). This was to separate children, adolescents, younger adults, and older adults because age is known to modulate olfactory function and play a role in recovery from olfactory dysfunction. The age ranges were based on previous experience [13,14,15,16] and on the need to consider the sample size of the investigated sample.

### 2.5. Psychophysical Olfactory Tests

Orthonasal olfactory function was evaluated in adults aged 18 years and older using the “Sniffin’ Sticks” test [13,17] (Burghart Messtechnik GmbH, Holm, Germany), comprised of odor threshold (T) and 16-item odor identification (I) tests. Children also underwent odor threshold testing using the “Sniffin’ Sticks” test. However, the 12-item “U-Sniff” odor identification test [15,16] (Burghart Messtechnik GmbH, Holm, Germany) was used as an age-appropriate odor identification test. The sum of both the threshold and the identification scores was regarded as the composite TI score (range: 1–32 in adults and 1–28 in children). Psychophysical olfactory testing was carried out once, after COVID-19 infection.

### 2.6. Cognitive Symptoms and Parosmia

Participants were asked about having difficulty in concentrating, memory, starting a task, or if they were experiencing “mental void” (as if the mind was blank). Patients were asked about the quality of their olfaction and those who reported a difference in smell perception were noted to have parosmia.

### 2.7. Data Analysis

SPSS software (IBM Corp. Released 2021. IBM SPSS Statistics for Windows, Version 28.0. Armonk, NY, USA: IBM Corporation) was used for the statistical analysis of the data. Some participants were excluded from the analyses due to incomplete data.

Analysis of variance (ANOVA), two-tailed independent sample *t*-tests, and odds ratios were computed for the data. One-way ANOVA between subjects was calculated to analyze differences between “recovery classes” and “age groups”. Independent *t*-tests were used to analyze the differences between dichotomous variables. Odds ratios were calculated to analyze the likelihood of “incomplete recovery” in those with parosmia. A *p*-value of <0.05 was considered significant, with a confidence interval of 95%.

## 3. Results

**Figure 1:**
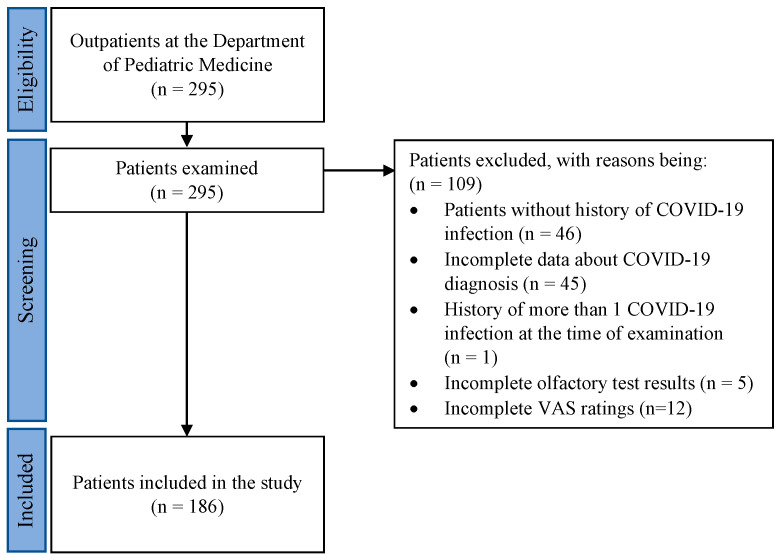
Patient selection flow chart, including inclusion and exclusion criteria.

A total of 186 patients (68 males, 116 females, 2 with sex undisclosed) between the ages of 5 and 62 years (mean age, 34 years) were included in this study (Figure 1).

The number of days between the date of evaluation and the COVID-19 diagnosis was between 22 and 820 days (mean interval, 396 days). There were no significant differences between VAS ratings and olfactory scores between males and females. The mean olfactory scores are illustrated in Table 1.

### 3.1. Recovery Classes

**Figure 2:**
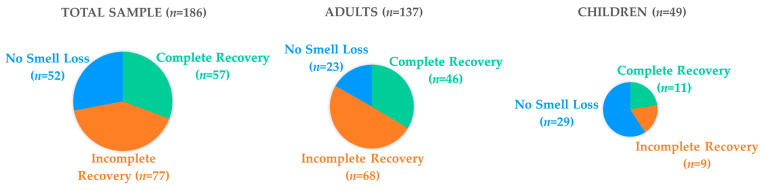
Subjective perception of recovery (“Recovery Classes”) percentages. “Complete Recovery” (in green), “Incomplete Recovery” (in orange), and “No smell loss” (in blue) for the entire population (Total Sample), for adults, and for children, respectively.

The characteristics of the three recovery classes are illustrated in Table 1 and Figure 2. With a mean interval between COVID-19 infection and the olfactory assessment of more than a year (393 days), the percentage of patients who still reported "incomplete recovery” was 42%. Furthermore, most adults reported “incomplete recovery” (*n* = 68, 49%), while this number was lower in children (*n* = 9, 18%). In contrast, 17% of adults (*n* = 23) had “no smell loss”, while this percentage was higher in children (*n* = 29, 57%).

**Figure 3:**
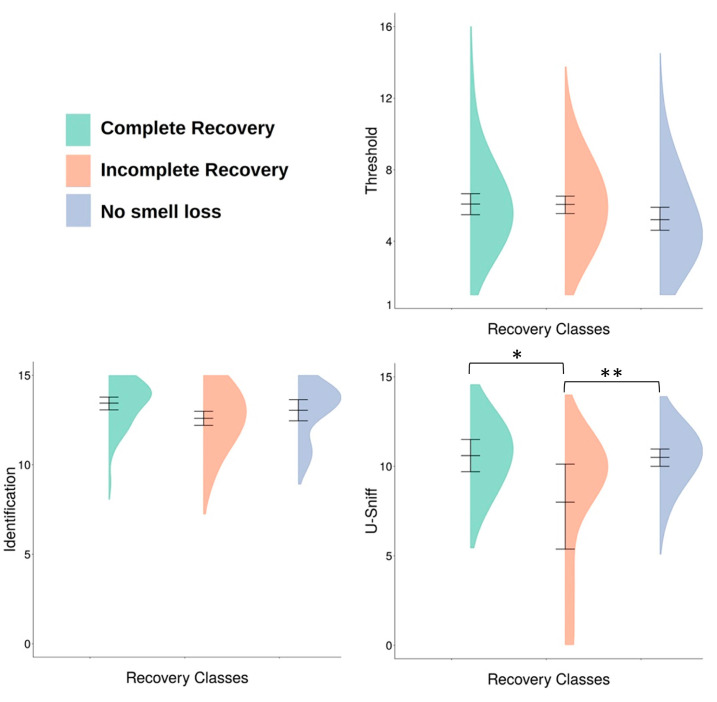
Patients’ mean threshold, identification, and U-Sniff scores with 95% confidence interval and their distribution among the three different “recovery classes” (“Complete Recovery” in green, “Incomplete Recovery” in orange, “No Smell Loss” in blue); * *p* = 0.03, ** *p* = 0.02.

There was no significant difference in threshold and adult identification scores between the three recovery classes (Figure 3). On the other hand, there was a significant difference in identification scores among the three recovery classes for children (F_2,46_ = 4.92, *p* = 0.02). Those with “incomplete recovery” had significantly lower U-Sniff scores compared to those with “complete recovery” (*p* = 0.03) and those with “no smell loss” (*p* = 0.02) (Figure 3). There were no significant differences in identification scores between comparisons of all other recovery classes. There were no significant differences in composite TI scores between recovery classes in children or in adults.

### 3.2. Subjective Olfactory Dysfunction and Recovery

**Figure 4:**
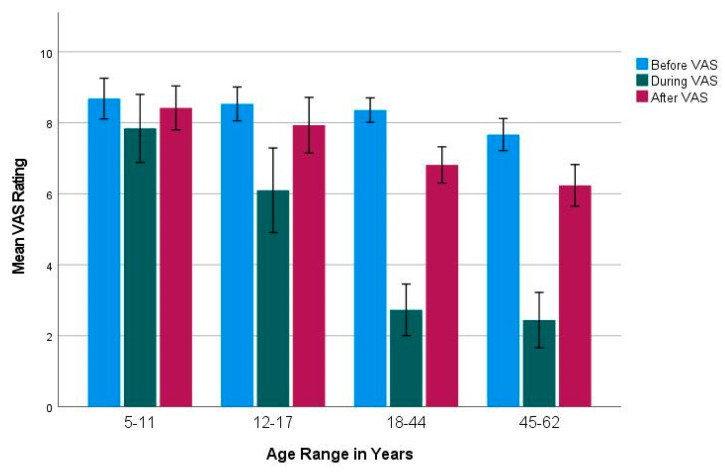
Mean VAS ratings for before (blue), during (green), and after (red) COVID-19 infection. The number of participants per age group (*n*): 5–11 years = 19; 12–17 years = 30; 18–44 years = 78; and 45–62 = 59.

The pediatric age groups had significantly higher VAS ratings during COVID-19 compared to those aged 18–44 and 45–62 years (F_1,182_ = 23.77, *p* < 0.001). The pediatric age groups also had significantly higher VAS ratings after COVID-19 (F_1,182_ = 7.22, *p* < 0.001) compared to those aged 18–44 (5–11 years: *p* = 0.03) and 45–62 (5–11 years: (*p* = 0.001) and 12–17 years: (*p* = 0.004)). There were no significant differences in VAS ratings between age groups before COVID-19 (Figure 4).

Adults reported a significantly greater decrease in VAS during COVID-19 compared to children (F_3,182_ = 15.22, *p* < 0.001), but a greater increase in VAS was also reported by adults after COVID-19 (F_3,182_ = 9.42, *p* < 0.001). When looking at the difference between VAS ratings before and after COVID-19, there was no significant difference between children and adults.

### 3.3. Cognitive Symptoms and Parosmia

Those who reported difficulty concentrating had lower VAS ratings during (t_153_ = 2.99, *p* = 0.003) and after (t_147.84_ = 2.68, *p* = 0.01) COVID-19 infection. They also reported a larger decline in VAS ratings for olfaction during COVID-19 (t_119.06_ = 2.53, *p* = 0.01) and greater difference in VAS ratings before and after COVID-19 (t_152.31_ = 2.53, *p* = 0.01) compared to those without difficulty concentrating.

Those with memory problems had higher threshold (t_158_ = −2.1, *p* = 0.04) and composite TI (t_158_ = −2.83, *p* = 0.01) scores and lower VAS ratings during (t_149.64_ = 3.05, *p* = 0.003) COVID-19 infection. They also reported a larger decline in VAS ratings for olfaction during COVID-19 (t_151_ = 2.91, *p* = 0.04) and greater increase after COVID-19 (t_151_ = −2.67, *p* = 0.01) compared to those without memory problems.

Those with “mental void” had a larger decline in VAS ratings for olfaction during COVID-19 (t_153_ = 2.35, *p* = 0.02) and greater difference in VAS ratings before and after COVID-19 (t_72.181_ = 2.5, *p* = 0.02) compared to those without “mental void”.

Those with difficulty in starting a task had greater difference in VAS ratings before and after COVID-19 (t_152.895_ = 2.75, *p* = 0.01).

Adults with parosmia had significantly lower odor identification test scores (t_114_ = 2.13, *p* = 0.04) and VAS ratings during (t_131.4_ = 5.51, *p* < 0.001) and after (t_151_ = 4.1, *p* < 0.001) COVID-19 infection. They also reported a larger decline in VAS ratings for olfaction during COVID-19 (t_101.02_ = 4.86, *p* < 0.001) and greater difference in VAS ratings before and after COVID-19 (t_63.25_ = 4.12, *p* < 0.001) compared to those without parosmia. Those with parosmia were also 3.5 times more likely to report incomplete olfactory recovery after COVID-19 (OR 3.5, 95% CI 1.65, 7.41).

## 4. Discussion

This study highlights three main findings: (1) compared to children, adults generally reported a greater decrease in olfactory function during COVID-19 infection but likewise also reported a greater increase afterwards; (2) although most adults reported “incomplete recovery” of olfactory dysfunction after COVID-19, there were no significant differences in measured olfaction between different recovery groups after COVID-19 infection; and (3) there were no significant differences between VAS ratings before and after COVID-19.

A study by Vaira et al. [18] found that although 66% of patients declared full recovery after resolution of COVID-19, 80% of these individuals still had hyposmia on psychophysical testing. In our study, although 41% reported incomplete olfactory recovery, there was no significant difference in threshold, identification (in adults), and composite TI scores between the different recovery classes. However, there was still a significant difference for odor identification in children, with those reporting “incomplete recovery” having the lowest scores, although the number of participants in this group was low.

Hence, the present study highlights that the subjective perception of olfactory loss from COVID-19 does not necessarily translate into a difference in psychophysically measured olfactory function. This may reflect an overestimation of olfactory loss in COVID-19 when VAS is used for evaluation. In addition, it seems to indicate that self-ratings alone are not the best measure to estimate recovery from COVID-19-associated olfactory loss, as they may be subject to numerous biases. Overall, the present results suggest that the prognosis for olfactory improvement in COVID-19 is good, where even those reporting “incomplete recovery” scored in the same range as individuals who did not experience COVID-19-associated olfactory dysfunction.

Gözen et al. [19] found significant differences between discrimination and identification scores, but not threshold, when comparing COVID-19 patients with and without self-reported smell loss. Although a significant difference in olfactory measurement was only found for identification scores in children in our study, we also found a higher likelihood of reporting “incomplete recovery” in patients with parosmia. Although most patients regain normal smell function after COVID-19 based on psychophysical measurement, we must remember that there are currently no standardized, validated psychophysical measures for qualitative olfactory dysfunction, and the presence of parosmia may be the reason behind reports of “incomplete recovery” in the setting of normal psychophysical tests. Parosmia is frequent in COVID-19-associated olfactory loss. At around 1 year after COVID-19 infection, the prevalence of parosmia still ranges between 23 and 61% in the literature [20,21,22]. It is associated with difficulties in odor identification that typically are not paralleled by major changes in odor thresholds [23].

Our results also suggest that the sense of smell appears to be less affected in younger compared to older individuals with COVID-19. Although adults reported a greater decrease in olfactory function during COVID-19, they also reported greater magnitude of improvement compared to children. Indeed, several studies demonstrated lower prevalence and incidence of OD in children compared to adults [3,4]. This may be explained by results from Somekh et al. [24], who correlated the prevalence of olfactory and sensory impairment between children and adults with the different concentrations of nasal epithelial angiotensin-converting enzyme 2 (ACE2) expression. Varying symptom severity of COVID-19 in relation to age was also reported in a review by Malcangi et al. [25]. They proposed a number of explanations for the age-related differences in severity of COVID-19-related neurological events, including differences in the concentration of ACE2 and varying immune system responses, as well as pre-existing endothelial damage and impaired cell regeneration. Furthermore, a study by Buonsenso et al. [26] found that three months after COVID-19 infection, only 2% of children still declared subjective smell dysfunction, while McWilliams et al. [27] showed that 38% of adults report full recovery, 54% partial recovery, and 8% report no recovery two years after infection. The latter study also revealed that those who were over 40 years old at the time of infection had a lower rate of recovery. However, we did not observe significant differences in VAS ratings between the age groups when accounting for differences between VAS after and during or after and before COVID-19.

Limitations of this study are related to the retrospective recollection of VAS ratings before and during COVID-19 infection, lack of psychophysical olfactory testing before and during COVID-19 infection, and the bimodal distribution of participants. It would have been ideal to have asked participants to rate their sense of smell and undergo psychophysical olfactory testing before and during COVID-19 infection. However, one needs to keep in mind that the study was designed to investigate exactly the correlation between rated olfactory function before, during, and after COVID-19, and measured olfactory function at threshold and suprathreshold levels.

## 5. Conclusions

There were no significant differences in the threshold and adult identification scores for those reporting “complete recovery”, “incomplete recovery”, or “no smell loss” after COVID-19 infection. However, children with “incomplete recovery” had lower identification scores than those with “complete recovery” (*p* = 0.033) and those with “no smell loss” (*p* = 0.022). The pediatric age groups had significantly higher VAS ratings during and after COVID-19 compared to older participants. Older individuals experienced greater magnitude of changes in their sense of smell after COVID-19 infection, but those with parosmia were 3.5 times more likely to report “incomplete recovery” of olfaction after COVID-19. The general prognosis for olfactory recovery after COVID-19 is good but appears to be confounded by the presence of parosmia, leading patients to subjectively report incomplete olfactory recovery. Although it is important to monitor recovery using validated psychophysical olfactory tests, subjective measures of olfaction help to provide specific insight, especially for qualitative olfactory dysfunction.

## Figures and Tables

**Table 1:** Mean and standard deviation of the three recovery classes for odor threshold, U-Sniff, and identification scores.

Recovery Class	*n*	Mean Threshold (SD)	*n*	Mean U-Sniff (SD)	*n*	Mean Identification (SD)
Complete Recovery	57	6.00 (2.36)	11	10.64 ^a^ (1.43)	46	13.43 (1.29)
Incomplete Recovery	77	5.95 (2.28)	9	8.11 ^ab^ (3.66)	68	12.71 (1.77)
No Smell Loss	52	5.21 (2.24)	29	10.38 ^b^ (1.50)	23	12.96 (1.52)
**Total**	186	5.76 (2.31)	49	10.02 (2.19)	137	12.99 (1.61)

Significant differences for U-Sniff scores between the “recovery classes” are indicated by letters a and b: (a) between “complete recovery” and “incomplete recovery” groups in children (*p* = 0.03); and (b) between “incomplete recovery” and “no smell loss” groups in children (*p* = 0.02).

## Data Availability

The data that support the findings of this study are available from the corresponding author upon reasonable request.
